# The Radiostability of Meropenem Trihydrate in Solid State

**DOI:** 10.3390/molecules23112738

**Published:** 2018-10-23

**Authors:** Karolina Kilińska, Judyta Cielecka-Piontek, Robert Skibiński, Daria Szymanowska, Andrzej Miklaszewski, Waldemar Bednarski, Ewa Tykarska, Anna Stasiłowicz, Przemysław Zalewski

**Affiliations:** 1 Department of Pharmacognosy, Poznan University of Medical Sciences, Święcickiego 4, 60-781 Poznań, Poland; karolinakilinska.mail@gmail.com (K.K.); jpiontek@ump.edu.pl (J.C.-P.); stasilowicz.anna@gmail.com (A.S.); 2 Department of Medicinal Chemistry, Medical University of Lublin, Jaczewskiego 4, 20-090 Lublin, Poland; robertskibinski@umlub.pl; 3 Department of Biotechnology and Food Microbiology, Poznań University of Life Sciences, Wojska Polskiego 48, 60-627 Poznań, Poland; daria.szymanowska@up.poznan.pl; 4 Institute of Material Science and Engineering, Poznan University of Technology, Jana Pawła II 24, 60-965 Poznań, Poland; andrzej.miklaszewski@put.poznan.pl; 5 Institute of Molecular Physics, Polish Academy of Sciences, Smoluchowskiego 17, 60-179 Poznań, Poland; waldemar.bednarski@ifmpan.poznan.pl; 6 Department of Chemical Technology of Drugs, Poznan University of Medical Sciences, Grunwaldzka 6, 60-780 Poznan, Poland; etykarsk@ump.edu.pl

**Keywords:** meropenem, radiation sterilization, radiostability, Q-TOF, antimicrobial activity

## Abstract

The influence of ionising radiation on the physicochemical properties of meropenem trihydrate in solid state was studied for doses of e-beam radiation: 25 kGy and 400 kGy. In the first part of our studies, we evaluated the possibility of applying radiosterilization to obtain sterile meropenem. No changes for meropenem irradiated with a dose of 25 kGy, the dose required to attain sterility, was confirmed in the results of spectroscopic (FT-IR), thermal (DSC, TGA) and X-ray powder diffraction (XRPD) studies. The radiation dose of 25 kGy produces no more than about 1500 ppm of radical defects. The chromatographic studies of irradiated meropenem in solutions did not show any chemical degradation. Moreover, the antimicrobial activity of meropenem irradiated with the dose of 25 kGy was unchanged. Based on the received results, we can conclude that radiostelization is a promising, alternative method for obtaining sterile meropenem. In the second part of the research, meropenem was exposed to e-beam radiation at the 400 kGy dose rate. It was confirmed, that reducing of antimicrobial activity could be connected with the degradation of β-lactam ring and changes in the trans-hydroxyethyl group. Apart from chemical changes, changes in the physical stability of irradiated meropenem (400 kGy) was also observed.

## 1. Introduction

A sterile dosage form for parenteral administrated drugs is necessary for safe therapy. Depending on the susceptibility to degradation of the active ingredient, various methods of sterilization are used. Sterilization under conditions of elevated temperature and humidity can be applied for stable drugs. However, alternative methods should be chosen for active ingredients susceptible to degradation. The radiation sterilization and its impact on the properties of drugs have been described in the literature for almost 50 years. The first studies were focused on confirming the effectiveness and advantages of irradiation as a method of sterilization and establishing a standard dose for this process [1,2,3,4,5,6]. Whereas the majority of currently conducted research is aimed at verifying the possibility of using radiation for a specific type of drug-effect of this method on the physicochemical properties of the compound while maintaining an adequate level of the sterility [7,8,9,10,11]. The foundations for such a verification are radiolitic studies based on the use of high doses of ionising radiation and a number of advanced analytical methods to understand the process of potential changes in stability of the drug and identification of radiolysis products [12]. Applying this approach is an opportunity to introduce alternative methods of sterilization for drugs with special requirements—such as carbapenem antibiotics.

Meropenem trihydrate (MRP) is a synthetic compound from the class of carbapenems. MRP is administered as intravenous infusion and has antibacterial activity against Gram-negative and Gram-positive bacteria as well as anaerobic microorganisms such as *Pseudomonas aeruginosa*, *Escherichia coli*, *Klebsiella pneumoniae*, *Enterobacter species*, *Serratia marcescens*, *Streptococcus pyogenes*, *Streptococcus agalactiae* and *Citrobacter* sp. It is indicated for the treatment of serious bacterial infection including complicated skin infections, complicated intra-abdominal infections, urinary tract infections, severe pneumonia, broncho-pulmonary infections and bacterial meningitis [13]. MRP as other carbapenems is characterized by high susceptibility to degradation under the condition of elevated temperature and humidity, both in solution and in solid state. Consequently, such conditions can lead to degradation of its key element—the β-lactam ring, responsible for antibacterial activity of the compound [14,15]. For these reasons, possibilities offered by the radiation sterilization, which is conducted in a solid state, seems to be the most advantageous for MRP. To the best of our knowledge, radiostability of MRP after the recommended sterilization dose (25 kGy) [16] has not been studied by other authors. However, there is one publication focusing on using the electron spin resonance spectroscopy for identification gamma-irradiated MRP with a maximum dose of 15 kGy [17].

In this paper, the effect of electron-beam radiation on MRP in the solid state has been investigated. A standard recommended dose of irradiation (25 kGy) in the context of application area and higher radiation doses (50–400 kGy), have been applied to understand the process of potential changes in MRP after sterilization. 

## 2. Results and Discussion

Our research on the radiostability of meropenem is divided into two areas. The first one is significant due to the application of radiation sterilization; and the second one is important from a cognitive point of view, aimed at evaluating the structure of the resulting degradation products.

For MRP samples exposed to irradiation, all changes were investigated using analytical and biological methods. 

**Figure 1:**
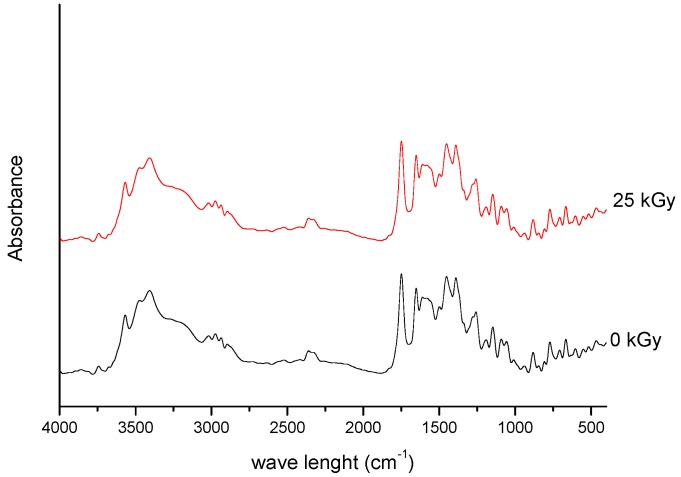
FT-IR spectra of unirradiated and irradiated (25 kGy) meropenem.

**Figure 2:**
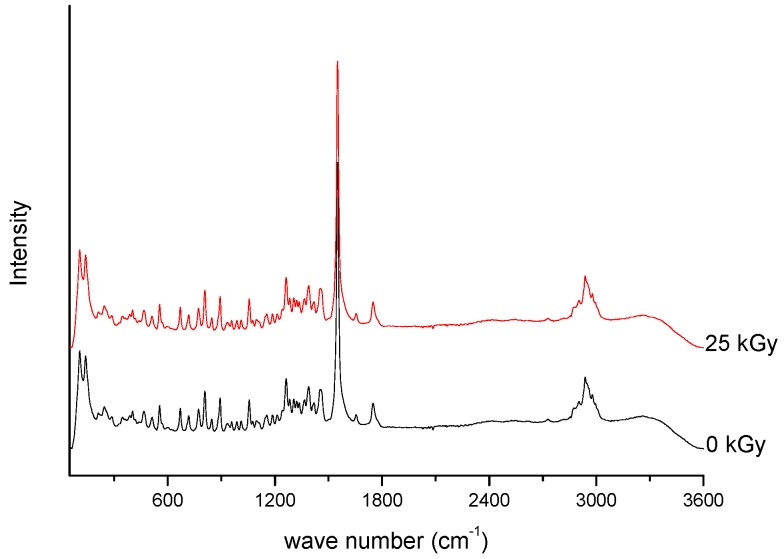
Raman spectra of unirradiated and irradiated (A-25 kGy) meropenem.

Spectroscopic spectra of irradiated (25 kGy) and non-irradiated MRP samples of MRP are presented in Figure 1 and Figure 2. 

For the 25 kGy irradiation dose, there were no changes in the position and intensity of the characteristic bands of meropenem. In our previous studies, we defined the characteristic bands of MRP based on the DFT (density functional theory) method [18], and then we analyzed the changes especially in relation to the bands corresponding to the labile bonds. The most significant bands that did not change their intensity and location are attributed to stretching and bending vibrations of O-H bonds in the carboxylic group, deformation vibrations of pyrrolidine ring, stretching and bending vibrations of the N-C bond in the β-lactam ring, stretching and scissoring vibrations of bonds in the dimethylcarbomoyl group, and stretching vibrations of C-H bonds in the hydroxyethyl group. Our observations confirmed the stability of meropenem at 25 kGy irradiation dose. To confirm these results, the study was continued by HPLC-MS/MS analysis. For samples irradiated at 25 kGy dose rate, the presence of degradation products was excluded. The literature data confirm that the most common problem encountered after radiosterilisation of solid drugs is discoloration or yellowing [19]. In our study, a colour change of samples after exposure to radiation was also reported—from white to slight pink. In the next step, we verified the impact of packing material on irradiated meropenem. None of the tested packages (PVP and glass containers) allowed to obtain sterile MRP without colour changes. What is particularly important, after dissolving the samples of irradiated MRP in water for injections, is that we got transparent and colorless solutions. Therefore, the authors tend to suggest that the colour change of the meropenem in solid state after exposure to irradiation, may be associated with the presence of the radiolyzed water trapped in the crystal structure of the meropenem.

**Figure 3:**
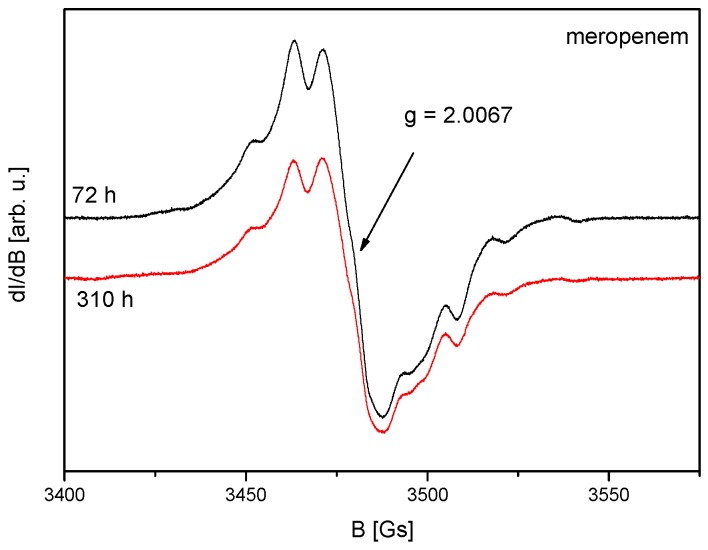
EPR spectra of irradiated meropenem recorded 72 h and 310 h after radiation sterilization (radiation dose 25 kGy).

Presented in Figure 3, EPR spectrum of e-beam irradiated MRP (dose 25 kGy) is very similar to that obtained after gamma-irradiation [17] and consists of many overlapping lines. 

**Figure 4:**
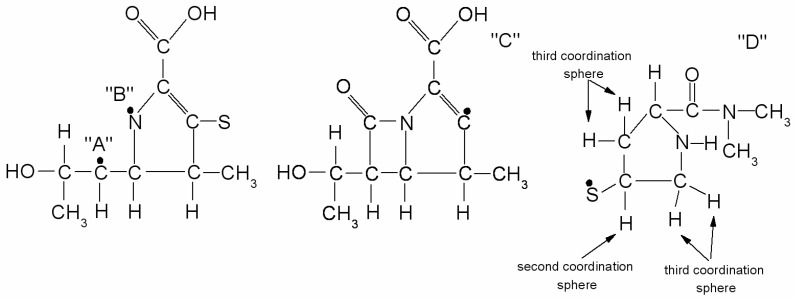
Four types of radicals (“A”, “B”, “C” and “D”) proposed in Reference [17] and created after gamma irradiation which were. Arrows point to protons from sulfur coordination spheres.

**Figure 5:**
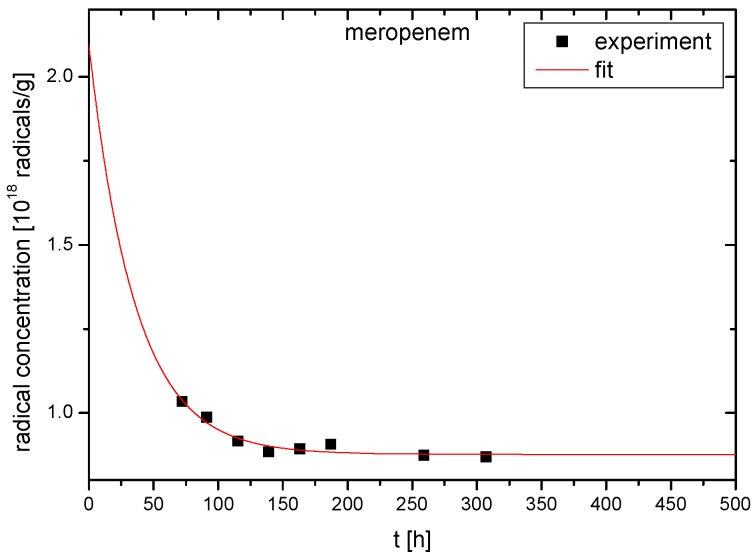
Concentration of free radicals vs. time after radiation sterilization (radiation dose 25 kGy).

In article [17], it was assumed that irradiation creates four types of radicals named as “A”, “B”, “C” and “D”, as presented in Figure 4. The authors of the above publication gave both spin Hamiltonian parameters and structural formulas of radicals. The proposed types of free radicals, however, seem very speculative. For example, in the radical “D”, an unpaired electron was founded mainly on sulfur. The hydrogen nuclei that can form the hyperfine structure of the EPR spectrum are found in the second (1 proton) and third (4 equivalent protons) coordination spheres of sulfur (Figure 4). It is, therefore, surprising why three different hyperfine constants from three single non-equivalent hydrogen nuclei were used to simulate the spectrum of the radical “D” [17]. To determine the type of radicals formed, the spectral resolution should be significantly increased by recording EPR spectra on a higher frequency (W or D bands). Unfortunately, our studies also do not provide information on the type of radicals formed, but indicate the number of defects caused by irradiation. The number of radicals decreases over time after irradiation, as presented in Figure 4 and Figure 5, and this indicates that not all radicals are stable at room temperature. 

**Figure 6:**
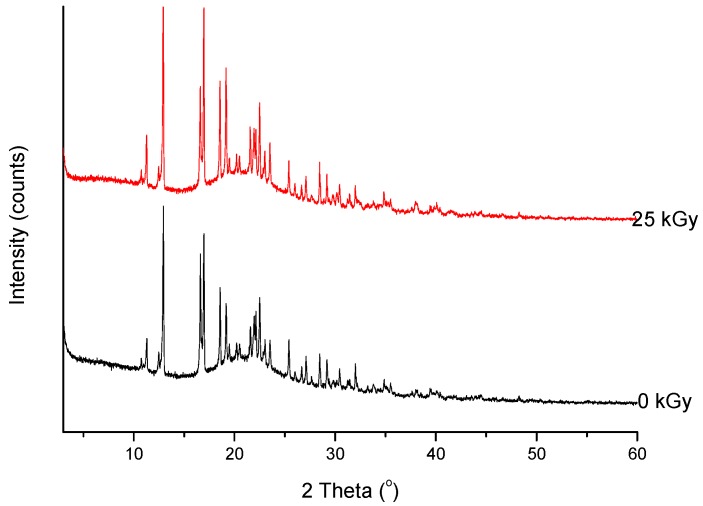
XRPD diffractograms of unirradiated and irradiated (25 kGy) meropenem.

To describe the total concentration of free radicals *I*(*t*) at any time *t* after irradiation we have used the formula:

(1) $$I(t)=I_{s}+I_{n}e^{\frac{-t}{T}}$$

where *I_s_* is the concentration of stable free radicals, *I_n_* is the concentration of unstable free radicals, and *T* is the mean lifetime of unstable radicals. The following parameters were obtained from fitting Equation (1) to experimental points: *I_s_* = 0.88(±0.01)⋅10^18^ radicals⋅g^−1^, *I_n_* = 1.20(±0.64)⋅10^18^ radicals⋅g^−1^, T = 36(±9) h. Since the highest concentration of free radicals occurs immediately after irradiation for *t* = 0 h and *I* (0) = *I_s_* + *I_n_* = 2.08(±0.64) × 10^18^ radicals⋅g^−1^, we can conclude that the radiation dose of 25 kGy produces no more than about 1500 ppm of radical defects. For non-irradiated MRP, the EPR signal was on the noise level and, therefore, the concentration of radicals was lower than about 3 ppm. The transient presence of free radicals (T = 36(±9) h.) did not lead to changes in the crystal structure of MRP (Figure 6). Moreover, free radicals did not induce changes in the microbial activity of meropenem. 

Analysis of changes of signals on the XRD diffractogram did not shown any shifts or changes in their intensity. Therefore, we would assume that any structural changes in the crystal lattice were not observed (Figure 6). 

**Figure 7:**
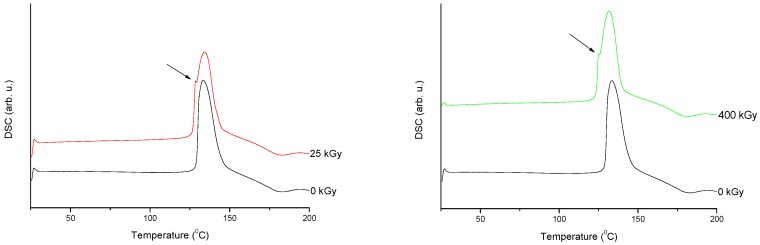
Differential scanning calorimetry (DSC) curves of non-irradiated and irradiated (A-25 kGy, B-400 kGy) meropenem. The arrows indicate the changes in the DSC spectrum after irradiation.

The DSC curves registered for irradiated and reference MRP samples are presented in Figure 7.

The thermodynamical parameters (melting enthalpies and characteristic temperatures) characterizing meropenem calculated from DSC data are summarized in Table 1.

For the MRP sample irradiated at the dose of 25 kGy, the thermograms shows only small changes in the peak position (ΔT_onset_ = 2.5 °C and ΔT_peak_ = 1.8 °C) in the comparison to the reference sample (0 kGy). The results of melting enthalpies are also very similar for both samples (ΔH = 296.6 J/g for non-irradiated sample and ΔH = 298.3 J/g for irradiated at 25 kGy dose rate). 

**Figure 8:**
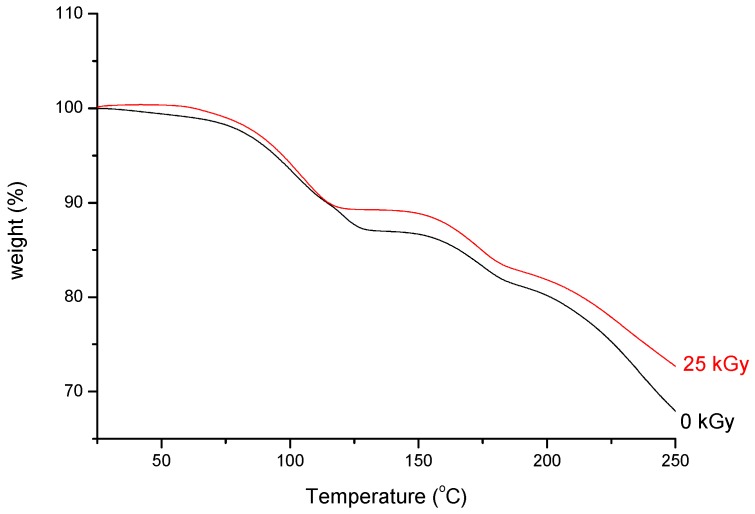
TGA curves of non-irradiated and irradiated (25 kGy) meropenem.

As can be seen from the TGA analysis (Figure 8), there are two stages in the thermal decomposition process. 

The first one corresponds to crystal water loss which is about 12.83% (0 kGy and 10.45% (25 kGy) when the temperature reaches 130 °C, which is nearly consistent with the calculated weight loss according to the stoichiometry of water in meropenem trihydrate (12.35%) and the second step from 150 °C with a weight loss because of the decomposition [20].

The antimicrobial activity of the irradiated (25 kGy) and non-irradiated samples of MRP was the same with regards to clinical isolates and references strains (Table 2).

**Figure 9:**
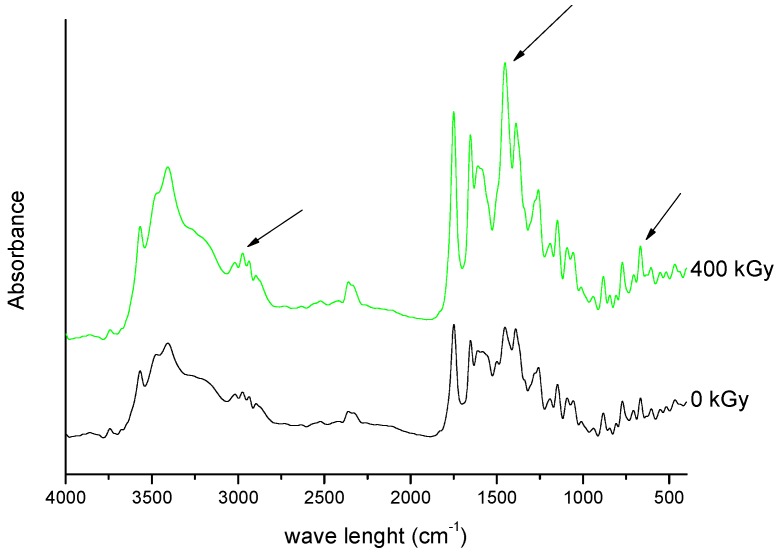
FT-IR spectra of unirradiated and irradiated (400 kGy) meropenem. The arrows indicate the changes in the FT-IR spectrum after irradiation.

**Figure 10:**
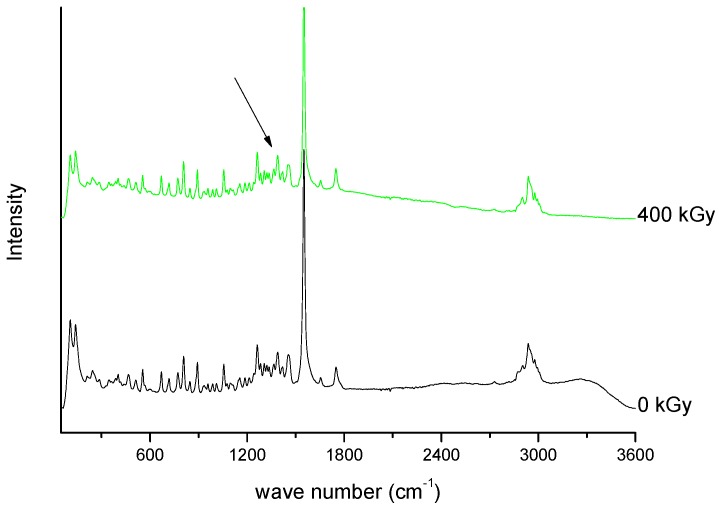
Raman spectra of unirradiated and irradiated (400 kGy) meropenem. The arrow indicates the change in the Raman spectrum after irradiation.

In the next part of our research, we evaluated the effect of a higher dose of irradiation (400 kGy). The identity evaluation in the solid state showed changes in the intensity of the band’s characteristics for molecular domains and changes in the crystallinity of samples. The analysis of FT-IR spectrum of irradiated MRP (Figure 9 and Figure 10) has shown that significant changes in the intensity of the characteristic bands of MRP. These changes were in the following ranges: 750–800 cm^−1^, 1250–1300 cm^−1^ and about 3000 cm^−1^, which corresponds to bending vibrations for the carbonyl bond in the carboxylic group, stretching vibrations of N-C bonds in structure β-lactam ring, and stretching vibrations for C-C bonds in the pyrrolidine ring, respectively. The changes observed in the range of 1580–1620 cm^−1^ on the Raman spectra were complementary to the changes observed in the FT-IR spectra of irradiated samples. These changes should be combined with a change in the intensity of vibrations of the C-H bond in the hydroxyethyl group.

Powder diffraction patterns exhibit a similar tendency to FT-IR spectra.

**Figure 11:**
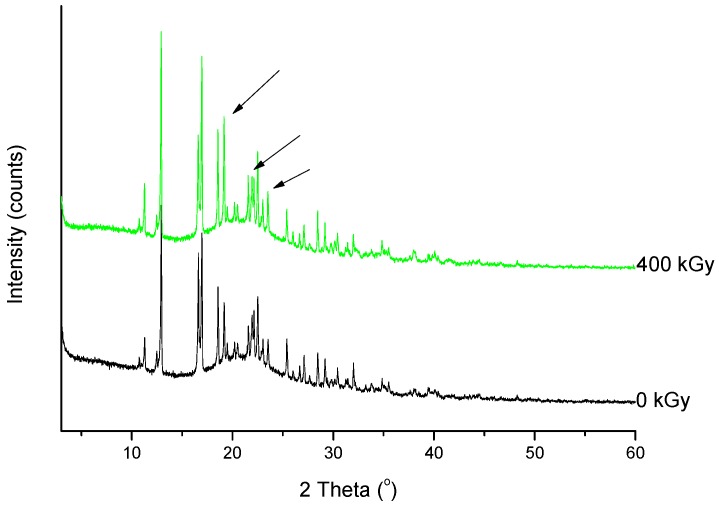
XRPD diffractograms of unirradiated and irradiated (400 kGy) meropenem. The arrows indicate the changes in the diffractogram after irradiation.

**Figure 12:**
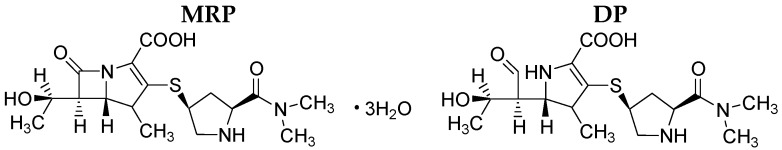
Structure of meropenem trihydrate (MRP) and its degradation product (DP).

**Figure 13:**
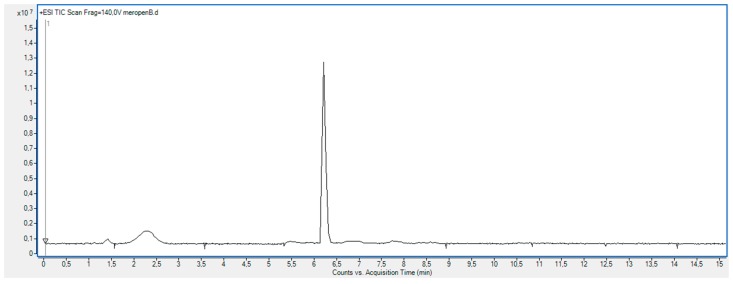
Total ion chromatogram (TIC) obtained under stress conditions (400 kGy) for meropenem trihydrate.

**Figure 14:**
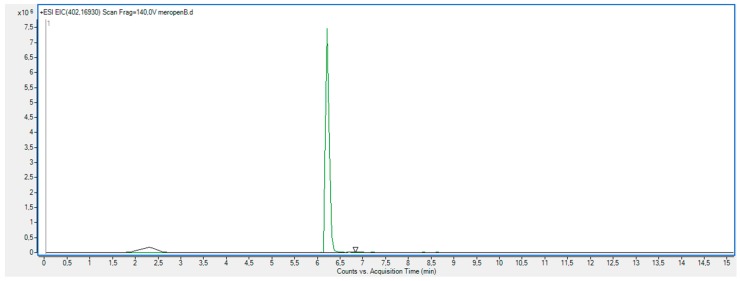
Extracted ion chromatogram (EIC) obtained under stress conditions (400 kGy) for meropenem (*m*/*z* 384.1588) and its degradation product M1 (*m*/*z* 402.1693).

**Figure 15:**
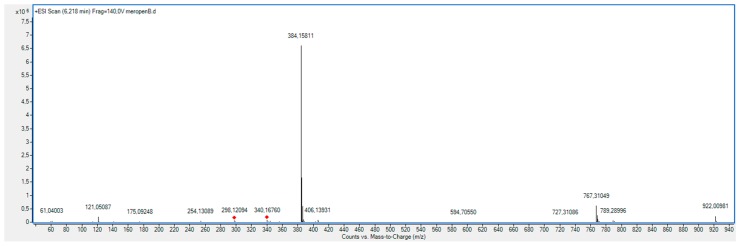
Full MS (TOF) spectra for meropenem (MRP).

**Figure 16:**
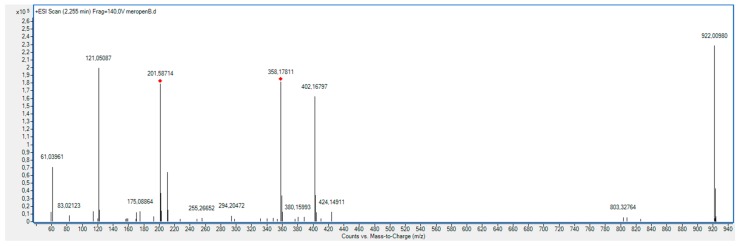
Full MS (TOF) spectra for degradation product (DP).

As shown in Figure 11, the XRD diffractogram of the polycrystalline MRP sample irradiated with a dose of 400 kGy, significantly differs from the others (0 kGy, 25 kGy), which indicates the structural changes occurring in the material at higher doses of radiation. The analysis of XRD diffractogram shows changes that seem to possibly suggest those related to the loss of water from the crystalline structure of the MRP. Beyond identifying possible physical changes in the structure of irradiated MRP samples, its chemical stability was tested. HPLC-DAD analysis [21] of irradiated samples showed a loss of content of 10%. The RSD between the three measurements was 1%. In our analysis of the resulting product (DP) (Table 3, Figure 12, Figure 13, Figure 14, Figure 15 and Figure 16), we pay particular attention to the degradation of the β-lactam ring. 

The degradation product (DP) is analogous to the structure of the product formed during acid-base hydrolysis (Figure 12) [22].

It should be emphasized that for β-lactam antibiotics, the presence of a ring determines bactericidal activity. Therefore, the authors connect the decrease in microbiological activity of irradiated MRP (400 kGy) against *Staphylococcus aureus* and *Acinetobacter baumannii* with the chemical disintegration of the β-lactam ring (Table 2). Authors also suggest that the decrease in microbiological activity of clinical isolates may be influenced by changes within the trans-hydroxyethyl group. The configuration of the trans-hydroxyethyl group is associated with the resistance of meropenem to selected bacterial strains (Table 2) [18].

## 3. Materials and Methods

### 3.1. Materials

Meropenem trihydrate was obtained from Chemos GmbH (Regenstauf, Germany). MRP is a white to light yellow crystal powder (purity > 98%), which is sparingly soluble in water and practically insoluble in ethanol and ether. All other chemicals and solvents were obtained from Merck KGaA (Darmstadt, Germany) and were of analytical grade. High quality pure water was prepared using the Millipore purification system (Millipore, Molsheim, France, model Exil SA 67120). 

### 3.2. Irradiation

The MRP were weighted into colourless glass vials (5 mg of each sample) and closed with plastic stoppers. Then vials were exposed to e-beam radiation at room temperature and air atmosphere in a linear electron accelerator LAE 13/9 (the dose 25 kGy per pass, 9.96 MeV electron beam and 6.2 μA current intensity) until the following doses were absorbed 25, 50, 100, 200 and 400 kGy.

### 3.3. Fourier Transform Infrared (FT-IR) Spectroscopy

Potential changes in the stability of irradiated and non-irradiated samples were examined by spectroscopy using Fourier Transform Infrared (FT-IR) spectrometer, IR Affinity-1 Shimadzu (Kyoto, Japan). Samples were prepared with potassium bromide as a matrix material, and were mixed in proportions of 1 mg of MRP sample to 300 mg KBr. The pressure of 15 tons/cm^2^ with a barrel of 13 mm in diameter was used to form pellets. Absorption spectra were measured with a resolution of 2 cm^−1^ within a wavenumber range from 4000 to 400 cm^−1^ with pure KBr pellet as a blank sample. For each spectrum, 30 scans were taken.

### 3.4. Raman Spectroscopy

The spectra were recorded on the FT-Raman module of a IFS66 FTIR/Raman spectrometer (Bruker Optics, Ettlingen, Germany). The setup consisted of a quartz beam-splitter and a Nd-YAG laser as the excitation source, which emits a continuous-wave laser energy at a wavelength of 1064.6 nm (9393.5 cm^−1^). The laser power was set to 300 mW. For each spectrum, 512 scans were taken with the resolution of 4 cm^−1^, in the range 3500–100 cm^−1^. All the measurements were conducted at room temperature. 

### 3.5. Electron Paramagnetic Resonance (EPR) Spectroscopy

A Bruker ELEXSYS 500 spectrometer (Bruker, Billerica, MA, USA) was used to detect the level of free radicals and to determine their concentration. Tests were carried out for irradiated and non-irradiated powdered samples of MRP in quartz capillaries (Wilmad) at a temperature of 24 °C and X-band microwaves (9.4 GHz). To avoid deformation of the signal by saturation effects, tests were conducted at low microwave power (2 mV) and spectra were recorded as a first derivative of the absorption signal. The calculations of the free radical numbers have been carried out using the method described by Mai et al. (2013) [23].

### 3.6. Differential Scanning Calorimetry (DSC)

Differential scanning calorimetry (DSC) data were collected by the use of a Phoenix DSC-204 (Netzsch) system at a scanning rate of 5 °C·min^−1^. The samples of about 3 mg of non- irradiated and irradiated meropenem samples were sealed in aluminium cells with pierced lids. The measurements were performed in a helium atmosphere within the temperature range from 25 °C to 200 °C. The results were processed using the TAA (Netzsch) software. The peak area was evaluated using linear baseline corrections.

### 3.7. Thermogravimetric Analysis (TGA)

Thermogravimetric analysis was performed on the Mettler-Toledo TGA 1/SF equipment which was calibrated before the experiment. Approximately 5 to 5.5 mg of the sample was put in an open aluminum oxide pan, and was heated at 10 °C·min^−1^ rates under 50 mL·min^−1^ nitrogen purge.

### 3.8. Microbiological Study

To verify the antimicrobial activity of MRP, the MIC (Minimal Inhibitory Concentration) values were determined for each reference strain from the American Type Culture Collection and clinical isolates. The experiments were run in triplicate. MICs for MRP were assayed using a serial dilutions method on the Mueller-Hinton liquid medium (Merck, Germany), which follows the standards of the Clinical and Laboratory Standards Institute (CLSI) [24]. In the tests, microbial culture with a standardized optical density was used.

### 3.9. X-ray Powder Diffraction (XRPD)

An X-ray powder diffraction (XRPD) pattern was obtained by means of a PANalitycal Empyrean system with Cu Kα_1_ radiation (1.54056 Å) at a voltage of 45 kV and a current 40 mA. The sample was scanned from 3° to 50° 2θ using a step size of 0.017° and the scanning rate 15 s/step with the sample spinning. The X-ray Diffractometer, D8 Advance (Bruker AXS, MA, USA) was used compared with Vario1 Johansson focusing monochromator and LynxEye linear detector.

### 3.10. HPLC-MS/MS Analysis

The analysis was conducted with use of an Agilent Accurate-Mass Q-TOF LC/MS G6520B system with a DESI ion source and an Infinity 1290 ultra-high-pressure liquid chromatography system consisting of a G4220A binary pump, a G1330B FC/ALS thermostat, a G4226A autosampler, a G4212A DAD and a G1316C TCC module (Agilent Technologies, Santa Clara, CA, USA). The control of the system, data acquisition and qualitative analysis were conducted with the use of the MassHunter workstation software B.04.00. Separations were performed on Poroshell, 2.7 µm particle size, 100 mm × 2.1 mm. The initial mobile phase composition was acetonitrile −0.1% formic acid (5:95) during 2 min. Then, gradient elution was used, starting from the mobile phase composition ratio (5:95) after 2 min to 30:70 within 15 min and the flow rate of the mobile phase was 0.3 mL min^−1^. The Q-TOF detector was tuned in the positive (4 GHz) and the main parameters were optimized as follows: drying gas 10L/min, nebulizer pressure 40 psig, gas temp. 300 °C, capillary voltage 3500 V, skimmer voltage 65 V, fragmentor voltage 200 V, octopole 1 radio frequency voltage 250 V. The data were acquired in the auto MS/MS mode with the mass range 50–950 *m*/*z* and the acquisition rate 1.2 spectra/s (for MS and MS/MS data). The collision energy was calculated from the formula 2 V (slope)*(*m*/*z*)/100 + 6 V (offset) and a maximum of two precursors per cycle were selected with an active exclusion mode after one spectrum for 0.2 min. To ensure the accuracy of the measurements, the reference mass correction was used and values of 121.0508 and 922.0097 *m*/*z* were selected as lock masses.

### 3.11. HPLC-DAD Analysis

The Dionex Ultimate 3000 analytical system consisted of a quaternary pump; an autosampler, a column oven and a diode array detector were used. The Kinetex, C18, 100A, 100 × 2.1 mm column (Phenomenex, Torrance, CA, USA) 5 µm particle sizes were used. The mobile phase was acetonitrile and 12 mmol·L^−1^ ammonium acetate (7:93). The flow rate of the mobile phase was 1.0 mL·min^−1^. The wavelength of the DAD detector was set to 298 nm. Separation was performed at 30 °C [21].

## 4. Conclusions

The irradiation dose recommended by the pharmacopoeia to obtain a sterile drug form (25 kGy) does not change the physical and chemical properties of the meropenem trihydrate, which makes the technique of radiation sterilization a promising alternative for current sterilization methods. Regarding the cognitive effect of exposure to radiation at a dose of 400 kGy, the following should be demonstrated as the most important factors: Creation of a product with an open β-lactam ring identical to the product of hydrolysis; changes in the crystal structure; decrease in activity against specific bacterial strains associated with disintegration of the β-lactam ring; and changes in the trans-hydroxyethyl group.

## Figures and Tables

**Table 1:** Melting enthalpies and characteristic temperatures of meropenem from DSC data.

(kGy)	T_onset_ (°C)	T_endset_ (°C)	ΔH (J/g)
0	129.9	145.9	296.6
25	127.4	144.1	298.3
400	124.4	141.2	288.8

**Table 2:** MIC values (mg·L^−1^) of meropenem and irradiated meropenem samples.

Microorganism Reference Stains or Clinical Isolates *	0 kGy	25 kGy	400 kGy
	mg/L		
*Salmonella enteritidis* *	250	250	250
*Salmonella enteritidis* ATCC 13076	32	32	32
*Salmonella typhimurium* *	250	250	250
*Salmonella typhimurium* ATCC 14028	32	32	32
*Staphylococcus aureus* *	**2**	**2**	**8**
***Staphylococcus aureus* ATCC 25923**	**0.25**	**0.25**	**2**
*Klebsiella pneumonia* *	250	250	250
*Klebsiella pneumonia* ATCC 31488	125	125	125
*Escherichia coli* *	8	8	8
*Escherichia coli* ATCC 25922	4	4	4
*Pseudomonas aeruginosa* *	16	16	16
*Pseudomonas aeruginosa* ATCC 27853	16	16	16
*Proteus vulgaris* *	0.5	0.5	0.5
*Proteus vulgaris* ATCC 8427	0.12	0.12	0.12
*Clostridium butyricum* *	16	16	16
*Clostridium butyricum* ATCC 860	16	16	16
*Clostridium pasterianum* *	16	16	8
*Clostridium pasterianum* ATCC 6013	0.25	0.25	0.25
*Acinetobacter baumannii* *	**32**	**32**	**64**
***Acinetobacter baumannii* ATCC 19606**	**8**	**8**	**16**
*Enterobacter aerogene s* *	250	250	250
*Enteroblacter aerogenes* ATCC 13048	125	125	250

**Table 3:** Q-TOF accurate mass elemental composition and MS/MS fragmentation of the analyzed substances.

Name	Retention Time	Measured Mass (*m*/*z*)	Theoretical Mass (*m*/*z*)	Mass Error (ppm)	Molecular Formula [M + H^+^]	MS/MS Fragmentation Ions (*m*/*z*)	MS/MS Fragment Formula
MRP	6.1	384.1573	384.1588	3.87	C_17_H_26_N_3_O_5_S	340.1668 298.1204 254.1304 141.1009 114.0360 68.0492	C_16_H_26_N_3_O_3_S C_13_H_20_N_3_O_3_S C_12_H_20_N_3_OS C_7_H_13_N_2_O C_5_H_8_NS C_4_H_6_N
DP	2.2	402.1670	402.1693	5.78	C_17_H_28_N_3_O_6_S	358.1771 216.0684 175.0883 140.1050 122.0946 96.0801	C_16_H_28_N_3_O_4_S C_16_H_28_N_3_O_4_S C_9_H_14_NO_3_S C_8_H_14_NO C_8_H_12_N C_6_H_10_N
